# Symposium 'methodology in medical education research' organised by the Methodology in Medical Education Research Committee of the German Society of Medical Education May, 25^th^ to 26^th^ 2013 at Charité, Berlin

**DOI:** 10.3205/zma000945

**Published:** 2015-02-11

**Authors:** Katrin Schüttpelz-Brauns, Claudia Kiessling, Olaf Ahlers, Wolf E. Hautz

**Affiliations:** 1 Medical Faculty Mannheim of Heidelberg University, University Medicine Mannheim (UMM), Department of Undergraduate Education and Educational Development, Mannheim, Germany; 2 Klinikum der Universität München, Institut für Didaktik und Ausbildungsforschung in der Medizin, München, Germany; 3 Charité - Universitätsmedizin Berlin, Department for Curriculum Management and Department for Anaesthesiology and Intensive Care Medicine CVK/CCM, Berlin, Germany; 4 Charité Universitätsmedizin Berlin, Department of Anaesthesiology and Intensive Care Medicine CVK/CCM, Berlin, Germany

**Keywords:** Methods of educational research, methodological qualification, continuous education

## Abstract

In 2013, the Methodology in Medical Education Research Committee ran a symposium on “Research in Medical Education” as part of its ongoing faculty development activities. The symposium aimed to introduce to participants educational research methods with a specific focus on research in medical education. Thirty-five participants were able to choose from workshops covering qualitative methods, quantitative methods and scientific writing throughout the one and a half days. The symposium’s evaluation showed participant satisfaction with the format as well as suggestions for future improvement. Consequently, the committee will offer the symposium again in a modified form in proximity to the next annual Congress of the German Society of Medical Education.

## Zusammenfassung

Im Rahmen seiner Qualifizierungsarbeit hat der Ausschuss für Methodik der Ausbildungsforschung im Jahr 2013 direkt im Anschluss an die Konferenz „Research in Medical Education“ ein Symposium ausgerichtet, dessen Ziel eine Einführung in die Methoden der Bildungsforschung unter spezieller Berücksichtigung der medizinischen Ausbildungsforschung war. Insgesamt 35 Teilnehmer konnten über eineinhalb Tage aufeinander aufbauende Workshops zu den Themen „qualitative Methoden“, „quantitative Methoden“ oder „Veröffentlichungen“ besuchen. Die Evaluation des Symposiums zeigte große Zustimmung zu dem Format, aber auch Ansatzpunkte für Verbesserungen in der Zukunft. Als eine Konsequenz wird der Ausschuss das Symposium in modifizierter Form in zeitlicher Nähe zur nächsten Jahrestagung der Gesellschaft für Medizinische Ausbildung erneut anbieten.

## Introduction

The Methodology in Medical Education Research Committee (MAF) of the Gesellschaft für Medizinische Ausbildung (Association of Medical Education in German-speaking countries, GMA) was founded in 2005 to improve the quality of studies (articles and contributions to congresses) as well as enhance specific research competencies [1]. 

Although many members of GMA have repeatedly stressed the need for face-to-face interventions, an internet search using Google for workshops and educational activities in this area did not yield results specific to medicine. An online survey among members of the Association for Medical Education in Europe (AMEE) also demonstrated a lack of methodological tools for medical education research [2]. The basics of medical education research are only taught within the scope of masters studies in medical education (MME). At the annual meetings of GMA, the committee was able to offer single separate workshops. Those were however limited in time due to the meeting’s overall schedule. Furthermore these workshops conflicted with the other duties of GMA members. Members of the committee recognized a major need for qualifications especially in medical education research methods such as selection and operationalization of appropriate dependent variables, recruitment of samples and sample size calculation, and development of methodological approaches for concrete examples.

The MAF committee therefore ran a symposium on methods for medical education research on May 25^th^/26^th^ 2013 at the “Lernzentrum” of Charité Universitätsmedizin Berlin. The event was designed for people who carry out medical education research and wish to gain the required methodological competence in theory and practice. To enable the participation of as many interested persons as possible the symposium was conducted directly after the Congress on Research in Medical Education (RIME) in Berlin. The symposium aimed to introduce participants to medical education research in the context of other disciplines of educational research and pedagogy. It furthermore aimed to provide opportunities to exchange experiences and make new contacts.

## Educational research in other disciplines

Three keynotes from educational researchers from different disciplines opened the symposium. The first lecture explored how statistical information may be presented to be understood, using the example of risk communication. The opportunity for everyone to understand and interpret statistical information was presented based on empirical research [3]. Originally developed to communicate risks to patients, the presented principles may be used in different contexts such as the development of educational material for students or publications for a larger public.

The second keynote introduced basic principles from cognitive load theory (CLT) for the design of didactical interfaces to e-learning applications [4]. The theory may not only be applied to the design of electronic learning platforms but is also relevant to the creation of powerpoint presentations and other educational material. The quality of materials used is thus important to the conduction of instructional interventions and materials should therefore be designed with careful attention to aspects of CLT. 

The third lecture provided an overview of concepts in evaluation [5], extending the term beyond routinely conducted measurements of student satisfaction following educational events. 

Evaluation refers to the quality of structure, process and results of projects and programs and should employ common methods of empirical social research. In doing so, evaluation or accompanying research in medical education may benefit from common concepts and methods in empirical social research. 

## Workshops in research methodology

**Figure 1:**
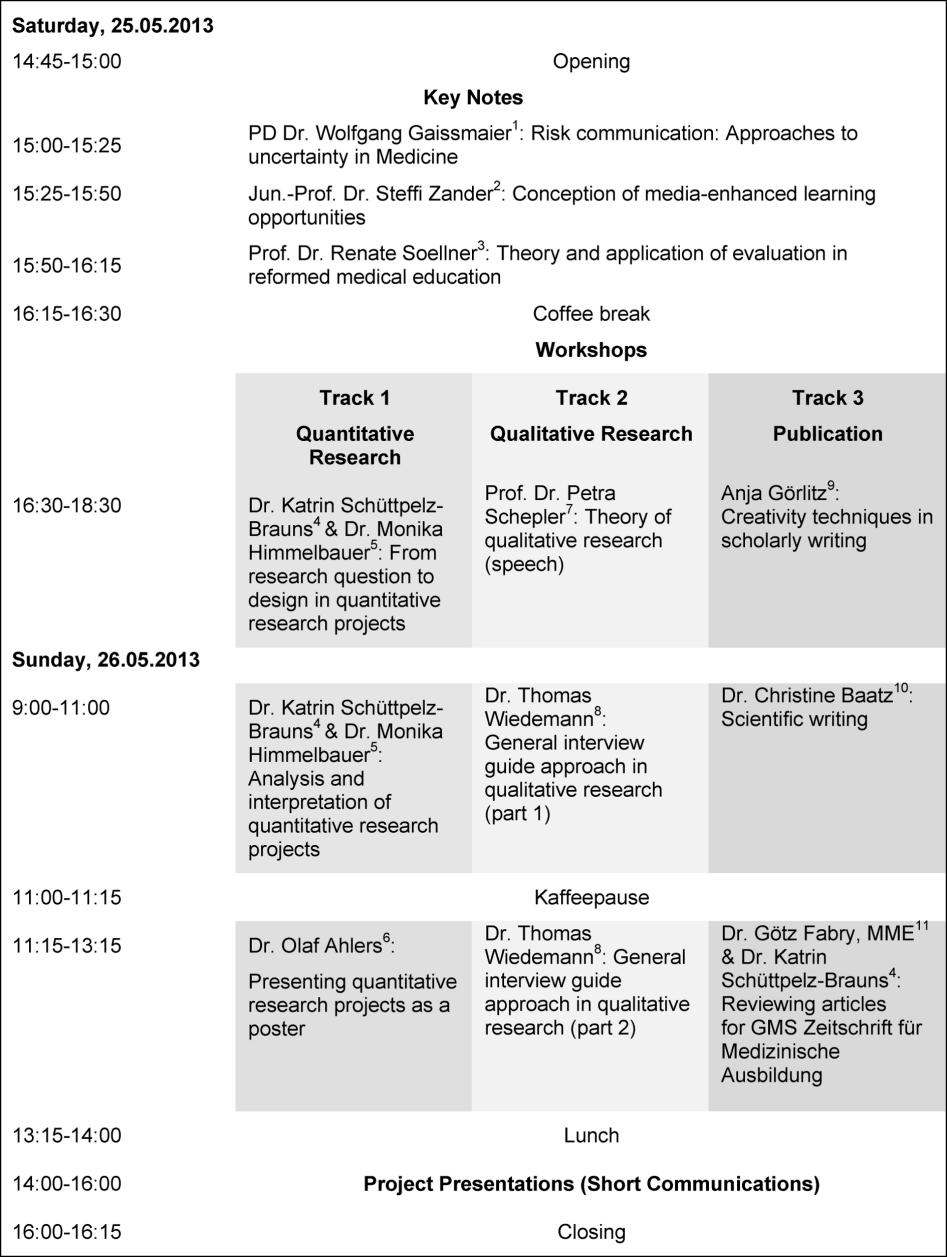
Program of the Symposium “Methods in medical education research” 2013

Three different workshop-tracks, each containing three workshops lasting two hours, were provided following the introductory key notes (see Figure 1 (Fig. 1)). Participants in track 1 used fictional examples of quantitative research projects to explore the development of a research question through research design and analysis up to the creation of a scientific poster in a hands-on manner [6], [7]. Track 2 was concerned with qualitative methods. The first part in this track introduced the theoretical basics of this area of research. Through practical examples, participants were introduced to the general interview guide approach in parts two and three [8], [9]. Track 3 on dissemination included training in creative writing techniques as well as in scientific writing. In cooperation with the editors of the GMS journal for medical education the third part of this track also introduced how to review manuscripts [10], [11], [12]. 

Workshops were centrally conceptualized and contained short didactic inputs and many practical examples, with the opportunity for exchange of ideas and experience, and discussion in small groups. 

## Discussion of examples from best practice

Different projects in medical education research were presented and discussed as best practice examples by the symposium’s participants in a plenary session following the workshops. 

Figure 1 (Fig. 1) presents an overview of the schedule that may also be accessed on 

http://aco.charite.de/forschung_entwicklung/symposium_methodik_der_ausbildungsforschung in a detailed form.

## Final considerations

The symposium for the first time presented an overarching concept to embed methodological and practical aspects in medical education research within educational research and pedagogy. The 35 participants had the opportunity to intensely engage in educational research from more than one perspective throughout 1.5 days. Because of the structure of the workshops provided (basic and advanced level), participants found competent partners to network with throughout the breaks. Key notes, workshops and project presentations oriented on practice were designed to not only convey knowledge and abilities but also provide ideas for their own projects and research.

An extensive evaluation of the symposium demonstrated that participants were heterogeneous in their backgrounds as the organisers intended and were satisfied with the format. 

Keynotes, workshops, presentations and discussion of practical examples were well evaluated overall. Participants asked for more time for discussion and exchange. Furthermore, they expressed a need for similar future events. Based on the positive experience of coupling the symposium to another event with similar audience, the next symposium will be conducted right before the annual GMA meeting without any overlap. Keynotes presenting the theoretical basics of educational research will be linked more closely to practice, thus aiming to demonstrate the application of empirical results in practice in successful models of adult education. Workshops will be restructured based on the evaluation and provide considerably more time while retaining their format. Project presentations will be transformed into a critical friends approach. Such a concept will be developed by the committee for methods in educational research.

Feedback demonstrated that the investment in time, money and manpower was well worth it. We hope that the format of the symposium in its next slightly adapted form will aid more researchers in medical education. 

## Acknowledgement

We thank Charité-Universitätsmedizin Berlin for making this symposium possible. We would further like to thank staff and student tutors at the “Lernzentrum” for their sustained efforts and contribution. 

## Competing interests

The authors declare that they have no competing interests.
